# Good practices according to WHO’s recommendation for normal labor and birth and women’s assessment of the care received: the “birth in Brazil” national research study, 2011/2012

**DOI:** 10.1186/s12978-016-0233-x

**Published:** 2016-10-17

**Authors:** Marcia Leonardi Baldisserotto, Mariza Miranda Theme Filha, Silvana Granado Nogueira da Gama

**Affiliations:** National School of Public Health, Oswaldo Cruz Foundation, Rio de Janeiro, Brazil

**Keywords:** Intrapartal care, Quality of care, Childbirth, Assessment of childbirth care

## Abstract

**Background:**

The World Health Organization recommends good practices for the conduct of uncomplicated labor and birth, with the aim of improving the quality of and assessment by women of childbirth care. The aim of this study was to evaluate the association between adoption of good practices according to WHO’s recommendation for normal labor and birth and assessment by women of the care received.

**Methods:**

Birth in Brazil is a national hospital-based study with countrywide representation consisting of 23,894 mothers and their newborns, conducted between February 2011 and October 2012. The present study analysed a subsample of this national survey. Postpartum women classified as low risk during pregnancy who had experienced either spontaneous or induced labor were included in this study, totalling 4102 mothers. To estimate the association between assessment by women of the childbirth care received (dependent variable) and good practices according to WHO’s recommendation during normal labor and birth (independent variables), a multinomial logistic regression analysis was used and crude and adjusted odds ratios calculated with their 95 % confidence intervals.

**Results:**

The good practices associated with positive assessment of the care received by women during labor and birth included the partner’s presence, privacy in the birthing place, time available to ask questions, clarity of information received, and empathic support from caregivers during labor and birth. Freedom of movement, free nutrition offered, choice of companions, nonpharmacological analgesia, skin-to-skin contact and breastfeeding in the childbirth room were not associated with the assessment by women of the care received.

**Conclusions:**

Our findings reveal the importance to mothers of their relationship with the team of caregivers during labor and birth. Therefore, caregiver teams must be qualified within a more humanistic vision of childbirth health care.

**Electronic supplementary material:**

The online version of this article (doi:10.1186/s12978-016-0233-x) contains supplementary material, which is available to authorized users.

## Background

Women’s assessment of the care received during labor and birth is an important component in the process of evaluation of the quality of care. Therefore, studies that aim to assess the patient’s views are necessary for monitoring and improving the quality of childbirth care offered [1, 2].

Positive assessment by women of the care received during labor and birth is associated with positive outcomes in the physical and mental health of the mother and infant, such as increased breastfeeding rate, better bonding between mother and newborn, and lower rates of future abortion. However, a negative assessment is associated with unfavorable outcomes such as psychological problems in the postpartum period (postnatal depression and post-traumatic stress disorder), preference for cesarean section, negative feelings and thoughts about the infant, and breastfeeding problems [3–5]. For these reasons, the women’s assessment about the childbirth care provided have been increasingly considered important feedback for policy makers, managers, and other professionals involved in maternal health care [1, 6].

The assessment by women of the care received is associated with the process and procedures adopted in assistance as well as women’s social, economic, and subjective characteristics. Furthermore, expectations and feelings about pregnancy may influence the way puerperal women assess childbirth care received [7–9].

Since 1996, the WHO recommendations have been published in the guide “Care in Normal Birth: A Practical Guide”, a series of practices and procedures to be adopted or avoided in the conduction of normal labor and birth for providing quality childbirth assistance. WHO has classified these practices into four categories according to their usefulness, effectiveness, and risks, based on the opinion of expert groups and according to the best scientific evidence. Category A comprises those obstetric practices which are demonstrably useful and should be encouraged; category B includes practices which are harmful or clearly ineffective and should be eliminated. Category C includes practices with insufficient evidence to support a clear recommendation that should be used with caution while further research is conducted. Category D practices are those that are often used inappropriately [10].

With the aim to improve care during labor and birth in Brazil, in the 2011 the Ministry of Health (MoH) launched a new program which included a number of actions to be taken within the National Health System to ensure that women have access to labor and childbirth assistance from the perspective of humanization. The changes in the childbirth care model proposed were based on two pillars: dignified and respectful treatment of women, their families, and the newborn; and adoption of good practices recommended by the World Health Organization (WHO) known to be beneficial for monitoring normal labor and birth [11].

The aim of this study is to evaluate the association between adoption of good practices in care during normal labor and birth, as recommended by WHO (category A) and assessment by women of the care received, using data from a nationwide survey and research.

## Methods

This study is an under sample of the larger study “Birth in Brazil”, a national hospital-based research with countrywide representation consisting of 23,894 mothers and their newborns, conducted between February 2011 and October 2012 in Brazil. All the authors were part of the team that conducted this survey and research. The sampling was carried out considering three stages of selection: all hospitals which had 500 or more births per year in 2007 were selected, classified according to Brazil’s five macro-regions (North, Northeast, Southeast, South and Mid-west), municipality (capital or interior), and type of hospital (private, public and mixed). Subsequently the number of days needed to reach the fixed sample of 90 women who had recently given birth in each hospital was calculated. Finally, these 90 women were selected from each hospital remaining in the sample. A total of 1356 (5.7 %) postnatal women selected were replaced, 203 owing to early hospital discharge and 1153 owing to refusal to participate. A detailed description of the “Birth in Brazil” methodology is given elsewhere [12].

### Sample subjects

To assess the outcome of interest (i.e., assessment by women of the care received during labor and birth), only postpartum women classified as low risk during pregnancy who had experienced either spontaneous or induced labor and whose birth had occurred in the Southeast region of Brazil were included. This geographical delimitation has been chosen because the Southeast has the highest prevalence of adoption of the good practices in care during normal labor and birth recommended by WHO [13]. Women were defined as low-risk according to the following criteria used by Dahlen et al. [14]: absence of pre-existing or pregnancy-related hypertension or diabetes; body mass index <30 (above which the person is considered obese); HIV negative; gestational age between 37 and 41 weeks; singleton pregnancy with cephalic presentation and birth weight between 2500 and 4499 g (between the 5th and 95th centiles of birth weight for gestational age). This resulted in a sample of 4102 mothers, representing 64 % of the total sample in the region [15].

### Data collection

A structured electronic questionnaire was administered face-to-face to women within the first 24 h after birth in the maternity ward querying their sociodemographic characteristics, obstetric history, prenatal care, and data related to labor and birth. In addition, medical record data of the mother and newborn were collected, and a photocopy made of the women’s prenatal care cards. Electronic forms were developed and validated to collect data and all interviews were conducted by interviewers previously trained by the investigation coordinators. Field research supervisors reapplied the questionnaire to a random sample of 5 % in the interviews with the women. Manuals were prepared with descriptions of procedures for data collection in order to ensure the quality of data and thereby minimize systematic or random errors.

Two telephone contacts were made with the mothers on average 45 days and 6 months after birth respectively, and structured questionnaires were applied at these moments. At the first telephone contact the women were asked about the presence of some WHO’s good practices recommended in care during normal labor and birth. At the second telephone contact they were asked about their assessment of the care received during labor and birth.

As it was not possible to contact all the women during the follow-up (68 % response rate in the first interview and 49.4 % in the second), a statistical model was adjusted to estimate the probability that each woman who took part at baseline would answer the telephone questionnaire, using a set of variables which differentiated the groups of respondents and non-respondents. Non-response adjustment factors attempt to compensate for the tendency of women to have certain characteristics (such as being unmarried or of lower education background) to respond at lower rates. On the basis of this model, specific sample weights were calculated for the analysis of the telephone interviews. The rationale for applying non-response weights is the assumption that non-respondents would have provided similar answers, on average, to respondents’ answers. More information about the sample design, data collection, and processing of lost segments is described elsewhere [12].

#### Study variables

The dependent variable of this study was the assessment by the women about the care received during labor and birth measured in the second telephone interview when they were asked: “In your opinion, how was the care that you received during labor and birth?” The answers were: 1) Excellent, 2) Good, 3) Regular 4) Poor, and 5) Very poor. Because of low frequencies for the categories poor and very poor, these were grouped into a single category called “Poor”.

The independent variables analysed were the good practices in care during normal labor and birth recommended by WHO (category A). They were obtained from the questionnaire administered to postpartum women in the hospital, medical record data, and the first telephone interview. Using these instruments, only some good practices could be analyzed: respecting the right of women to privacy in the birthing place, empathic support from caregivers during labor and birth, respecting women’s choice of companions during labor and birth, presence of companion throughout labor and birth, giving women as much information and explanation as they desired (time to ask questions and receive information), clarity of the information and explanation received, offering oral fluids and food during labor and birth (free nutrition), nonpharmacological pain relief during labor, freedom of position and movement throughout labor, early skin-to-skin contact between mother and child, and support for the initiation of breastfeeding in the birthing place [16].

The control variables used were parity (primiparous or multiparous), type of birth (vaginal, vaginal with use of forceps or vacuum extractor, and cesarean section), type of payment (public or private with payment by the patient or by health insurance), educational level (0–7, 8–10, 11–14, and 15 or more years) and economic level. According to the Brazilian Association of Research Companies (ABEP), the definition of economic level used in this study was based on the ownership of assets and education level of the head of household [15]. The categories of economic level were divided into five groups, ranging from A (highest) to E (lowest). Because of the low proportion of women in classes A and E, the categories were regrouped into three levels: A and B (high), C (mid level), D and E (low).

#### Data analysis

For this study, exploratory and descriptive data analysis were conducted first. After this, bivariate and multivariate analyses using the generalized linear modeling technique of multinomial logistic regression were conducted and Odds ratios (OR), crude and adjusted for potential confounding variables, and 95 % confidence intervals (CI) were obtained. These measures were used to assess the associations between the dependent and independent variables. For data analysis, R version 3.0 software (The R Foundation, Vienna, Austria) and IBM SPSS version 19.0 (IBM Corp., Armonk, NY, USA) were used.

## Results

Table 1 shows the women’s assessment of the care received, as well as sociodemographic and obstetric variables. The majority of women were age 20–34 years old (71 %), belonged to the lower middle economic class (60.5 % in class C), had between 11 and 14 years of education (43.7 %), and identified (self-reported) as mixed skin color (54.8 %). About 90 % of women had their births financed by the public sector. With respect to parity, almost half of the sample was primiparous (49.1 %). Vaginal delivery and cesarean section had 73.5 and 22.3 % prevalence, respectively. Regarding assessment of care during labor and birth, 37.3 % of women rated the care received as excellent, 52.1 % as good, 7.2 % as regular, and 3.4 % as poor or very poor.

**Table 1:** Proportion of assessment of the care received, socio-demographic and obstetric characteristics of postpartum women

	Number	Percent
Assessment		
Excellent	1529	37.3
Good	2137	52.1
Regular	310	7.2
Poor/Very poor	126	3.4
Parity		
Multiparous	2114	50.9
Primiparous	2041	49.1
Type of birth		
Vaginal	3055	73.5
Forceps/Vacuum Extractor	173	4.2
Caesarean section	928	22.3
Age (years)		
12–19	952	22.9
20–34	2947	71.0
35 or more	255	6.1
Skin color		
White	1416	34.1
Black	397	9.6
Mixed	2275	54.8
East Asian	66	1.1
Educational level (years)		
15 or more	180	4.4
11 to 14	1809	43.7
8 to 10	1259	30.4
0 to 7	891	21.5
Economic level		
High (A + B)	886	21.5
Mid level (C)	2501	60.5
Low (D + E)	741	18.0
Type of payment		
Private	466	11.2
Public	3689	88.8

Concerning the prevalence of good practices in childbirth care, about a quarter of the women had a companion present during labor and birth, and in 90.7 % of cases the companion was the free choice of the women. An offer of free nutrition was reported by 34.5 % of participants, and 45 % were able to move about freely. Prevalence of the use of nonpharmacological methods for pain relief was 37.5 %. Skin-to-skin contact with the newborn 34.1 % and breastfeeding in the childbirth room 48.6 % of women were reported (Table 2).

**Table 2:** Prevalence of good practices in normal labor and birth (WHO)

Good practices WHO	Excellent		Good		Regular		Poor		Very poor	
	n	%	n	%	n	%	n	%	n	%
Empathic support from caregivers	1751	42.1	1868	44.9	352	8.6	68	1.6	116	2.8
Privacy in birthing place	1717	41.3	1955	47.1	323	7.8	75	1.7	85	2.1
Clarity of the information/explanation	1589	38.2	1880	45.2	448	10.8	124	3.1	114	2.7
Receiving information/explanation	1139	27.4	2230	53.7	546	13.1	140	3.4	101	2.4
	Yes		No							
Women’s choice of companions	2907	90.7	298	9.3						
Free nutricion	1412	34.5	2744	65.5						
Freedom position/movement	1871	45.0	2285	55.0						
Use of non-pharmacological methods	2618	37.5	1538	62.5						
Skin to skin contact	1413	34.1	2732	65.9						
Initiationof breast-feeding in birthing place	2010	48.6	2139	51.6						
	No		Partial		Yes					
Presence of companion	962	23.1	2192	52.8	1008	24.1				

This study found that 90 % of women assessed as excellent or good several aspects of their relationship with the team of caregivers, such as privacy in the birthing place, the empathic support of professionals, clarity of information received, and time to ask the staff questions. Approximately 2.5 % assessed each of these aspects as very poor (Table 2).

In the adjusted multinomial model, the variables of empathic support and respectful health professionals, privacy in the birthing place, clarity of information received by the woman, time available to ask questions and receive explanations, and presence of a companion during labor and childbirth were associated positively and significantly with assessment by mothers of the care received during labor and birth (Table 3).

**Table 3:** *Odds ratio* (OR) crude and adjusted^a^ of good practices in normal labour and birth (WHO) with the assessment by women of the care received

Good practices WHO	Women’s assessment of the care received^b^					
	Good		Regular		Poor/Very poor	
	OR crude (CI)	OR adjusted (CI)	OR crude (CI)	OR adjusted (CI)	OR crude (CI)	OR adjusted (CI)
Empathic support from caregivers						
Excellent	1.00	1.00	1.00	1.00	1.00	1.00
Good	4.13 (2.91–5.87)	4.03 (2.90–5.59)	3.15 (1.43 6.90)	3.31 (1.48–7.39)	11.17 (3.06 40.82)	10.77 (2.65–43.75)
Regular/Poor/Very poor	6.43 (3.72–11.13)	6.53 (3.73–11.42)	40.63 (19.38–85.18)	46.81 (20.65–106.12)	217.00 (59.71 788.65)	257.14 (66.22–998.46)
Privacy in birthing place						
Excellent	1.00	1.00	1.00	1.00	1.00	1.00
Good	4.03 (2.95–5.50)	3.87 (2.85–5.24)	7.59 (3.69–15.63)	8.56 (3.88–18.91)	16.38 (3.41–78.73)	16.97 (3.54–81.29)
Regular/Poor/Very poor	5.21 (2.78–9.76)	5.16 (2.73–9.74)	44.56 (19.57–101.48)	55.12 (21.88–138.83)	182.96 (30.95–1081.73)	217.18 (33.73–1398.43)
Clarity of the information/explanation						
Excellent	1.00	1.00	1.00	1.00	1.00	1.00
Good	3.64 (2.86–4.65)	3.44 (2.69–4.39)	3.39 (1.88–6.10)	3.38 (1.82–6.30)	12.31 (3.34–45.36)	10.96 (2.94–40.88)
Regular/Poor/Very poor	6.46 (3.42–12.21)	6.02 (3.29–10.99)	26.25 (14.40–47.86)	58.98 (15.10–55.66)	88.26 (21.47–362.83)	88.88 (21.45–368.24)
Receiving information/explanation						
Excellent	1.00	1.00	1.00	1.00	1.00	1.00
Good	3.04 (2.18–4.25)	2.93 (2.08–4.12)	5.14 (1.97–13.44)	5.03 (1.90–13.32)	3.69 (1.48–9.21)	3.71 (1.49–9.28)
Regular/Poor/Very poor	6.17 (4.19–9.07)	5.78 (3.95–8.46)	33.63 (13.71–82.47)	33.77 (13.08–87.19)	23.27 (8.27–65.42)	25.74 (8.79–75.32)
Free nutricion						
Yes	1.00	1.00	1.00	1.00	1.00	1.00
No	0.96 (0.62–1.42)	1.00 (0.66–1.53)	0.88 (0.57–1.35)	0.89 (0.54–1.45)	0.81 (0.39–1.68)	0.76 (0.35–1.64)
Freedom position/movement						
Yes	1.00	1.00	1.00	1.00	1.00	1.00
No	0.87 (0.60–1.25)	0.95 (0.65–1.38)	0.92 (0.59–1.43)	0.98 (0.62–1.55)	0.59 (0.32–1.11)	0.61 (0.32–1.16)
Use of non-pharmacological methods						
Yes	1.00	1.00	1.00	1.00	1.00	1.00
No	0.83 (0.61–1.12)	0.87 (0.63–1.20)	1.18 (0.72–1.95)	1.24 (0.74–2.07)	0.97 (0.49–1.93)	0.99 (0.51–1.91)
Skin to skin contact						
Yes	1.00	1.00	1.00	1.00	1.00	1.00
No	1.28 (0.85–1.93)	1.29 (0.84–1.99)	1.25 (0.63–2.47)	1.26 (0.61–2.60)	2.28 (0.97–5.35)	2.28 (0.93–5.56)
Initiation of breast-feeding in birthing place						
Yes	1.00	1.00	1.00	1.00	1.00	1.00
No	0.93 (0.69–1.25)	0.99 (0.72–1.35)	1.08 (0.59–1.97)	1.10 (0.59–2.06)	0.81 (0.44–1.47)	0.78 (0.43–1.42)
Women’s choice of companions						
Yes	1.00	1.00	1.00	1.00	1.00	1.00
No	1.36 (0.81–2.28)	1.28 (0.76–2.15)	0.98 (0.25–3.86)	0.83 (0.22–3.08)	2.52 (0.86–7.35)	2.02 (0.64–6.41)
Presence of companion						
Yes	1.00	1.00	1.00	1.00	1.00	1.00
Partial	0.96 (0.61–1.51)	0.98 (0.61–1.57)	0.85 (0.37–1.92)	0.89 (0.41–1.92)	1.27 (0.47–3.41)	1.12 (0.40–3.14)
No	1.32 (0.77–2.26)	1.28 (0.72–2.30)	2.38 (0.88–6.43)	2.44 (0.99–6.03)	3.51 (1.07–11.50)	3.51 (1.11–11.12)

Because of the low proportions of mothers, the Regular, Poor and Very poor categories in all good practices were grouped

*CI* 95 % confidence interval

^a^Models adjusted for sociodemographic variables (economic level, education level, type of payment), parity and type of birth

^b^Reference category: Excellent

In addition, the better the opinion of each woman with respect to these practices, the more favorable her assessment of the care received. This pattern was seen for all variables related to the relationship with the caregiver team. Compared to women who had assessed empathic and respectful support from professionals as excellent, those who had assessed this variable as regular, poor, or very poor were approximately 47 times more likely (OR = 46.81, CI = 20.65–106.12) to rate their care as regular and 257 times more likely (OR = 257.14, CI = 66.22–998.46) to assess care as poor or very poor (Table 3).

Women who did not have a companion present during labor and birth had a 3.51 times greater likelihood (OR = 3.51, CI = 1:11–11:12) of assessing the care received as poor or very poor compared with those who had a companion with them at all times. The other examined good practices in category A (free nutrition, freedom of movement, nonpharmacological analgesia for pain, breastfeeding in the childbirth room, skin-to-skin contact after birth, and free choice of a companion) had no statistical association with the outcome (Table 3).

## Discussion

This study demonstrated that a good relationship established between women and their health care team during labor and birth is a decisive factor for positive assessment of the care received. The lack of association with more objective aspects of care, such as the carrying out of certain procedures, may be due to the fact that these have less relevance in the assessment process by mothers. Many studies have reported that more subjective aspects of care, usually related to how the medical team interacts with the mother, actually have more weight in positive assessment of care than practices related to the objectives of care procedures [16, 17].

In one systematic review, Hodnett [17] found four factors associated with satisfaction during childbirth: the pregnant woman’s expectations for childbirth, the quantity and quality of support received from the medical staff by the woman, the quality of the patient relationship with the medical team, and the woman’s involvement in decisions about childbirth. According to this author, these four factors seem to outweigh factors of age, socioeconomic status, skin color, birth preparedness, physical environment, pain, immobility, and medical interventions. Therefore, the behavior and the doctor and patient relationship seem to have more weight and are more associated with satisfaction than the other variables [17].

Corroborating with this line of reasoning, the information and explanations received during labor and birth are important for good assessment of care because these help with the woman’s autonomy related to childbirth. If a woman is not informed about the progress of her labor and any procedures that will be carried out, she cannot participate in the choices made, and therefore only receives passive childbirth care. Studies demonstrate that a feeling that women are protagonists during childbirth is associated with high levels of positive care assessment [18, 19].

A relevant aspect of this study is the importance of a companion’s presence throughout labor and birth for a positive evaluation of care. Others studies also report this factor as being significant for a perception of quality care by mothers [20–22].

Another important issue is the discrepancy founded between the low prevalence of good practices for normal labor and birth and the high prevalence of positive assessment by mothers of the care received. It is necessary to discuss the reasons that may lead women to positively assess childbirth care that is not in accordance with the ones recommended by the MoH and WHO. One of the possible causes for this gap would be the lack of information that pregnant women have about what constitutes quality assistance during childbirth. Studies have pointed out that in Southeaster Brazil, prenatal care does not provide information about good practices and the rights of women [23, 24]. Because of this, most women do not know their rights and the procedures that comprise optimal care. Therefore, it would not be possible for women to accurately assess their care if they are unfamiliar with what is considered to be good quality childbirth care. This fact has been pointed out as one of the limitations of this type of study, in which patients are asked to assess their care when they are unfamiliar with good standards of practice and care [6].

Added to the issue of women who are given insufficient information is their expectations about childbirth, which also influence how childbirth care is assessed [25, 26]. In their study of the women’s expectations and experiences related to childbirth, Dias and Deslandes [27] found reports of verbal abuse, abandonment, and delayed care, suggesting that many women classified the care received as good only because they did not experience any violence [27]. So it could be that the expectations of these women are so low that even when poor care is received, they will positively assess the assistance.

Another factor reported in the literature that may be associated with these discrepancy is the tendency for women to assess the care received during labor and childbirth more positively than it actually was [3]. Van Teijlingen et al. [6] called this trend “gratitude bias”. According to these authors, this bias permeates and hinders many studies that investigate the assessment and satisfaction of mothers with childbirth care received. These authors suggest that some women cannot negatively rate their care because they consider such an act to be ingratitude for the positive outcome of the childbirth [6].

A number of methodological issues need to be took in account before considering the implications of the results of this study. Firstly, due to the low prevalence of women in some categories of assessment of care received, some OR’s had a high confidence interval (CI), which compromises the accuracy of these measures of association. Secondly, the absence of any control variable related to the women’s expectations in relation to the care received during labor and childbirth made impossible an analysis in depth of the associations.

In spite of the above, the fact that care assessment was measured outside of the hospital and, on average, 1 year after childbirth, helped to reduce the gratitude bias and is a strength of our research. Because studies recommend that this type of question should be made postpartum after hospital discharge because women may feel embarrassed and afraid of reprisals from the health care team while still in the hospital. In addition, the critical sense of women in relation to assistance received during labor and birth tends to increase with time [3, 6]. Furthermore, as far as we know, this is the first study of its kind in Brazil.

## Conclusions

In this study, the way women assessed their care during labor and birth was influenced by good practices related to how they were treated by the medical team (privacy in the birthing place, time available to ask questions and receive explanations), clarity of information received, the empathic and respectful support of health care providers, and the presence of a companion during labor and birth. This result shows the importance of the relationship between the team of caregivers and the woman, for a positive experience of childbirth.

Our study did not find associations between care assessment and good practices related to the objective aspects of care: nonpharmacological analgesia, free nutrition, free movement, initiation of breastfeeding in the childbirth room, and skin-to-skin contact immediately after birth. This fact may be explained by the gratitude bias, the women’s lack of information, and low expectations for labor and birth assistance. However, we cannot ignore the possibility that perhaps the subjective aspects of childbirth care have more relevance to women, according to our findings and those of other researches.

The relationship between the caregiver team and the mother has great impact on the way she will experience the process of labor and birth. An attentive and welcoming staff, with good listening and communication skills, can help to improve the quality of care. Therefore, this study points to the need to invest in training health care professionals so as to improve these qualities and skills, in view of the goal to develop care guided by the concept of humanization, which respects the woman’s dignity, rights, and autonomy.

## Additional files

Additional file 1: Portuguese version of the article. (DOCX 107 kb)
Additional file 2: Peer review. (PDF 407 kb)

## Abbreviations

MoH: Brazil’s Ministry of Health
WHO: World Health Organization
HIV: Human Immunodeficiency Virus
ABEP: Brazilian Association of Research Companies
OR: Odds ratio
CI: Confidence interval
