# Supplementary material for: Good practices according to WHO’s recommendation for normal labor and birth and women’s assessment of the care received: the “birth in Brazil” national research study, 2011/2012
Source: Reprod Health. 2016 Oct 17;13(Suppl 3):124. doi: 10.1186/s12978-016-0233-x (PMC5073993; doi:10.1186/s12978-016-0233-x)
Supplement: Additional file 1: — Portuguese version of the article. (DOCX 107 kb) [file 12978_2016_233_MOESM1_ESM.docx]

**Adoção das boas práticas na assistência ao trabalho de parto e parto normais recomendadas pela OMS e avaliação realizada pelas puérperas sobre o cuidado recebido: pesquisa “Nascer no Brasil”, 2011-2012.**

Márcia Leonardi Baldisserotto,

Escola Nacional de Saúde Pública Sergio Arouca, Fundação Oswaldo Cruz.

[mlbaldisserotto@gmail.com](mailto:silvana.granado@gmail.com)

Mariza Miranda Theme Filha

Escola Nacional de Saúde Pública Sergio Arouca, Fundação Oswaldo Cruz.

marizatheme@hotmail.com

Silvana Granado Nogueira da Gama

Escola Nacional de Saúde Pública Sergio Arouca, Fundação Oswaldo Cruz.

[silvana.granado@gmail.com](mailto:silvana.granado@gmail.com)

**Autor para correspondência:**

Marcia Leonardi Baldisserotto

Escola Nacional de Saúde Pública – ENSP

Rua Leopoldo Bulhões 1480 sala 813

Manguinhos – Rio de Janeiro – Brasil

CEP: 20041-210

e-mail: [mlbaldisserotto@gmail.com](mailto:mlbaldisserotto@gmail.com)

**Resumo:**

Introdução: A Organização Mundial de Saúde recomenda a adoção de boas práticas para a condução do trabalho de parto e parto normais com o objetivo de melhorar não apenas a qualidade da assistência, mas também a avaliação feita pelas mulheres sobre este cuidado recebido no parto. O objetivo deste estudo foi avaliar a associação entre a adoção das boas práticas recomendadas pela OMS para a condução do trabalho de parto e parto normais e a avaliação realizada pelas mulheres sobre o cuidado recebido.

Método: O “Nascer no Brasil” é um estudo de base hospitalar, com representação nacional, constituído de uma amostra de 23.894 puérperas e seus recém-nascidos, realizado entre Fevereiro de 2011 e Outubro de 2012. Para este artigo, foi analisada uma subamostra desta pesquisa nacional, no qual foram incluídas apenas as puérperas classificadas como de baixo risco durante a gravidez, que entraram em trabalho de parto espontâneo ou induzido e que tiveram seus bebês na região sudeste. Desse recorte, resultou uma amostra de 4102 mães e seus conceptos. Para estimar a associação entre a avaliação realizada pelas mulheres sobre a assistência recebida durante o trabalho de parto e parto (variável dependente) e as boas práticas do grupo A recomendadas da OMS para condução do trabalho de parto e parto normais (variáveis independentes), foi utilizada a regressão logística multinomial. Dessa análise, foram obtidas as razões de chances (*Odds Ratio – OR*) brutas e ajustadas e seus intervalos de confiança de 95%.

Resultados: As boas práticas associadas com a avaliação positiva da assistência recebida pelas mulheres durante o trabalho de parto e nascimento foram a presença do acompanhante, privacidade no local do parto, tempo disponível para fazer perguntas, clareza das informações recebidas e apoio empático e respeitoso da equipe de cuidadores. As demais boas práticas: livre movimentação, dieta livre, companheiro ser de livre escolha da parturiente, analgesia por métodos não farmacológicos, contato pele-a-pele e início do aleitamento ainda na sala de parto não apresentaram associação com o desfecho.

Conclusões: Os resultados revelam a importância para as mulheres da qualidade do relacionamento entre elas e a equipe de cuidadores durante o trabalho de parto e parto. Portanto, as equipes de cuidadores devem ser qualificadas para estabelecerem uma relação de qualidade com a parturiente, visando, assim, uma assistência mais humanista.

Palavras-chave: cuidado intraparto; qualidade do cuidado; parto; avaliação da assistência ao trabalho de parto e parto.

**Introdução:**

A visão das puérperas a respeito do cuidado recebido durante o trabalho de parto e parto é um componente importante no processo de avaliação da qualidade da assistência prestada. Por esse motivo, estudos que visam mensurar a avaliação realizada pelo paciente do cuidado recebido são necessários para o monitoramento e aprimoramento da qualidade da assistência prestada durante o trabalho de parto e parto nas maternidades [1,2].

Trabalhos demonstram que uma avaliação positiva das mulheres sobre a assistência recebida durante o trabalho de parto e parto está relacionada a desfechos positivos na saúde física e psíquica da mãe e do bebê no puerpério, tais como: aumento da taxa de aleitamento materno, melhora da qualidade do vínculo mãe e bebê e menor taxa de aborto futuro. Em contrapartida, uma avaliação negativa é associada a desfechos negativos: problemas psíquicos no puerpério (depressão pós-parto e transtorno de estresse pós-traumático), preferência por partos cesarianos, sentimentos e pensamentos negativos a respeito da criança e maiores problemas em relação à amamentação [3-5]. Por causa disso, a avaliação realizada pelas puérperas do cuidado recebido durante o trabalho de parto e parto tem se tornado, cada vez mais, um importante “*feedback”* para formuladores de políticas públicas, gestores e demais profissionais [1,6].

Os fatores associados a uma boa avaliação da assistência recebida durante o trabalho de parto e parto dizem respeito não apenas às características do cuidado em si, mas também aos aspectos sociais, econômicos e subjetivos da mulher. Somado a isso, as expectativas e os sentimentos em relação à gestação também podem influenciar na forma como a puérpera avaliará o cuidado recebido [7-9].

A Organização Mundial de Saúde (OMS), desde 1996, preconiza, através do guia “*Care in normal birth: a pratical guide*”, uma série de práticas e procedimentos que devem ser adotados ou evitados na condução do trabalho de parto e parto normais para uma assistência de qualidade. A OMS classificou essas práticas em quatro categorias de acordo com sua utilidade, eficácia e periculosidade, baseada na opinião de especialistas sobre o tema e nas melhores evidências científicas disponíveis na época. Essas categorias são: categoria A - práticas demonstradamente úteis e que devem ser estimuladas, categoria B - práticas que são claramente prejudiciais ou ineficientes e devem ser eliminadas, categoria C - práticas sem evidência científica suficiente para serem recomendadas e que devem ser usadas com cautela enquanto não há outras evidências e categoria D - procedimentos utilizados frequentemente de forma inapropriada [10].

Com o objetivo de melhorar a assistência prestada no trabalho de parto e parto no Brasil, em 2011, o Ministério da Saúde (MS) lança o Projeto da Rede Cegonha, cujo conjunto de regras e medidas devem ser adotadas no Sistema Único de Saúde (SUS). Essa proposta de mudança do modelo de assistência ao parto é baseada em dois principais pilares: tratamento com dignidade e respeito à mulher, aos seus familiares e ao recém-nascido por parte dos profissionais de saúde e das instituições de modo geral e adoção das boas práticas sabidamente benéficas para o acompanhamento do parto e do nascimento preconizadas pela Organização Mundial de Saúde [11].

O objetivo deste estudo é avaliar a associação entre a adoção das boas práticas na assistência ao trabalho de parto e parto normais recomendadas pela OMS (categoria A) e a avaliação realizada pelas puérperas sobre o cuidado recebido a partir dos dados da pesquisa “*Nascer no Brasil*”.

Método:

Esse estudo utilizou uma sub-amostra da pesquisa *“Nascer no Brasil”,* um estudo de base hospitalar com representação nacional composto de 23.894 mães e seus conceptos realizado entre fevereiro de 2011 e outubro de 2012. Todos os autores fizeram parte da equipe que conduziu essa pesquisa. A amostragem foi realizada em três estágios. O primeiro correspondeu aos estabelecimentos de saúde, no qual foram selecionados hospitais que registraram 500 ou mais partos, segundo os dados do Sistema de Informações sobre Nascidos Vivos (SINASC) de 2007, e estratificados segundo as cinco macrorregiões do Brasil (Norte, Nordeste, Sudeste, Sul e Centro-oeste), a localização (interior e capital) e o tipo de estabelecimento (privado, misto e público). No segundo estágio, utilizou-se o método de amostragem inversa para calcular o número de dias necessários (no mínimo sete) para obtenção de uma amostra fixa de 90 puérperas para cada hospital. O terceiro estágio compreendeu as puérperas elegíveis que deram à luz a recém-nascido vivo, independente do peso ao nascer e idade gestacional, ou natimorto com peso ao nascer maior que 500g ou idade gestacional maior ou igual a 22 semanas. Um total de 1356 (5.7%) puérperas selecionadas foram substituídas, 203 devido à alta hospitalar precoce e 1153 à recusa em participar da pesquisa. Mais informações sobre o método de amostragem da pesquisa “*Nascer no Brasil”* encontram-se em outro artigo [12].

Sujeitos da amostra

Para análise do desfecho de interesse (avaliação realizada pelas puérperas sobre o cuidado recebido durante trabalho de parto e parto), foram incluídas somente as puérperas classificadas como gestantes de risco obstétrico habitual durante a gestação que entraram em trabalho de parto (espontâneo ou induzido) na região sudeste. Essa delimitação geográfica se deve ao fato do Sudeste apresentar a maior prevalência de adoção das boas práticas na assistência ao trabalho de parto e parto [13].

Definiu-se como gestante de risco habitual aquelas sem histórico de diabetes ou hipertensão arterial gestacional ou pré-gestacional, não obesas (IMC < 30), HIV negativas, com idade gestacional entre 37-41 semanas ao nascer, gravidez única, com feto em apresentação cefálica, com peso ao nascer entre 2.500g e 4.499g e entre o 5º e 95º percentil de peso ao nascer por idade gestacional [14]. Desse recorte, resultou uma amostra de 4102 puérperas, representando 64% do total da região.

Coleta de dados:

Um questionário eletrônico estruturado foi aplicado face a face, ainda na maternidade, às puérperas nas primeiras 24 horas após o parto. Através desse instrumento, foram coletadas as características sociodemográficas, os antecedentes obstétricos, as informações da assistência pré-natal e os dados relativos ao trabalho de parto e parto. Além disso, foram coletados dados do prontuário da mãe e do recém-nascido e fotografados os cartões de pré-natal das puérperas. Os formulários eletrônicos para a coleta de dados foram desenvolvidos e validados pela equipe da pesquisa, e todas as entrevistas foram realizadas por entrevistadores previamente treinados pelos coordenadores da pesquisa. A fim de garantir a qualidade dos dados e minimizar os erros sistemáticos ou aleatórios, os supervisores de campo da pesquisa reaplicaram o questionário a uma amostra aleatória de 5% nas entrevistas com as mulheres. Somado a isso, manuais com descrições de procedimentos para a recolha de dados foram preparados e utilizados pelos entrevistadores.

Dois contatos telefônicos foram feitos com as mães, em média, 45 dias e seis meses após o nascimento, respectivamente, e questionários estruturados foram aplicados nesses momentos. No primeiro contato telefônico, as mulheres foram questionadas sobre a presença de algumas das boas práticas recomendadas pela OMS no cuidado durante o parto e nascimento normais. No segundo contato telefônico, a mulher foi indagada sobre a sua avaliação do cuidado recebido durante trabalho de parto e parto.

Como não foi possível contatar todas as mulheres durante o follow-up (taxa de resposta de 68% na primeira entrevista e 49,4% no segundo), um modelo estatístico foi ajustado para estimar a probabilidade de cada mulher que participou na linha de base do estudo responder ao questionário por telefone, utilizando um conjunto de variáveis que diferenciava e caracterizava os grupos de respondentes e não-respondentes. Com base neste modelo, foram calculados pesos diferenciados para as respostas para cada um desses grupos. A justificativa para a aplicação desses pesos diferenciados é a suposição de que as mulheres dentro de um mesmo grupo com características semelhantes (respondentes e não-respondentes) teriam fornecido, em média, respostas semelhantes entre si. Mais informações sobre o desenho amostral, coleta de dados e processamento de segmentos perdidos é descrito em outro lugar [12].

Variáveis do estudo

A variável dependente (desfecho) do estudo foi a avaliação realizada pela puérpera sobre o cuidado recebido durante o trabalho de parto e parto mensurada na segunda entrevista telefônica quando foi perguntado: “Na sua opinião, o atendimento ao seu parto foi: 1. Excelente, 2. Bom, 3. Regular, 4. Ruim e 5. Péssimo”. Devido a baixas frequências das categorias “Ruim” e “Péssima”, as mesmas foram reagrupadas em uma única categoria denominada “Ruim”.

As variáveis independentes testadas foram aquelas que compõem a categoria A das boas práticas recomendadas pela OMS, que são consideradas úteis e que devem ser estimuladas, obtidas do questionário aplicado à puérpera no hospital, dos dados do prontuário e da primeira entrevista telefônica. Esses instrumentos possibilitaram analisar algumas das boas práticas desse grupo: privacidade no local do parto; apoio empático dos profissionais, clareza das informações recebidas, tempo disponível para obter informações e fazer perguntas, presença de acompanhante durante o trabalho de parto e parto, dieta livre, livre movimentação, analgesia por métodos não farmacológicos, contato pele a pele logo após o nascimento ainda na sala de parto e aleitamento materno na sala de parto [16].

As variáveis de controle utilizadas foram: parturição (primípara ou multípara), tipo de parto (vaginal, vaginal com uso de fórceps ou vácuo extrator e cesáreo), escolaridade (ensino fundamental incompleto; ensino fundamental completo, ensino médio completo e ensino superior completo/mais), tipo de financiamento do parto (público ou privado – pagamento direto pela puérpera ou plano de saúde) e condição econômica. A definição de condição econômica utilizada nesse estudo seguiu os critérios preconizados pela Associação Brasileira de Empresas de Pesquisa (ABEP), baseado na posse de bens e no grau de escolaridade do chefe da família [15]. As categorias de condição econômicas foram divididas em cinco faixas, variando de A (mais elevada) a E (mais baixa). Devido à pequena proporção de mulheres nas classes A e E, as categorias foram reagrupadas em três níveis: A e B (alta), C (média) D e E (baixa).

Análise dos dados:

Para este estudo, inicialmente, foram conduzidas análises exploratórias e descritivas dos dados. Posteriormente, realizou-se análises bivariadas e multivariadas através do modelo de regressão logística multinomial (GLM). Foram obtidas, através desses modelos logísticos multinomial, *Odds Ratio* (OR) brutos e ajustados para potenciais variáveis de confusão e seus intervalos de 95% de confiança. Essas medidas foram utilizadas para verificar as associações entre as variáveis dependentes e independentes. Para análise dos dados, foram utilizados o software R versão 3.0 (The Foundation R, Viena, Áustria) e o IBM SPSS versão 19.0 (IBM Corp., Armonk, NY, EUA).

Aspectos éticos:

Este estudo foi realizado segundo a resolução n^o^ 196/1996 do Conselho Nacional de Saúde, que orienta as normas de pesquisas com seres humanos, emitido pelo Comitê de Ética em Pesquisa da Escola Nacional de Saúde Pública Sérgio Arouca, sob o número CAAE 27754514.0.0000.5240. Um formulário eletrônico de consentimento livre e esclarecido, que incluiu autorização para a coleta de dados dos prontuários médicos da mãe e do recém-nascido, para fotocopiar cartões de pré-natal das mulheres e para os subsequentes contatos telefônicos, foi lido para os responsáveis de cada instituição e para todas as mulheres participantes. O consentimento era dado de forma verbal e todos os participantes receberam uma cópia impressa do formulário.

Resultados:

A Tabela 1 mostra a prevalência das avaliações realizadas pelas mulheres sobre o cuidado recebido durante o trabalho de parto e parto, bem como as variáveis sociodemográficas e obstétricas. A maior parte das puérperas entrevistadas tinham de 20-34 anos de idade (71%), pertenciam à classe econômica C (60,5%), possuíam ensino médio completo (43,7%) e se autodeclararam de cor de pele parda (54,8%). Cerca de 90% dessas mulheres tiveram seus partos financiados pelo setor público (Tabela 1).

Quanto à parturição, praticamente metade da amostra era composta de primíparas (49,1%). Das mulheres que entraram em trabalho de parto, 73,5% evoluíram para o parto vaginal e 22,3% para cesárea. Em relação à variável de desfecho (avaliação pela puérpera do cuidado recebido durante o trabalho de parto e parto), aproximadamente 90% das mulheres avaliaram positivamente o cuidado recebido: 52,1% como bom e 37,3% como excelente. Apenas 3,4% das mulheres classificaram a assistência como ruim ou péssima. A categoria de avaliação regular foi referida por 7,2% das puérperas (Tabela 1).

Em relação à prevalência das boas práticas na assistência ao trabalho de parto e parto, observou-se que um quarto das mulheres tiveram acompanhante durante todo o trabalho de parto e parto, sendo que, em 90.7% desses casos, a companhia foi de livre escolha da mulher. A livre dieta foi reportada por 34.5% das participantes e 45% puderam movimentar-se livremente. Houve adoção dos métodos não farmacológicos de alívio para dor para 37.5% das parturientes. O contato pele a pele com o RN ocorreu em 34.1% dos casos e 48.6% iniciaram a amamentação ainda na sala de parto (Tabela 2).

Referente às boas práticas de caráter mais subjetivas, que dizem respeito, em grande parte, ao comportamento e à relação da equipe de cuidadores com a parturiente, tais como privacidade no local do parto, apoio empático dos profissionais, clareza nas informações e tempo para fazer perguntas, aproximadamente 90% das mulheres avaliaram como excelente ou boa cada um desses aspectos da assistência. Uma parcela de cerca de 3,5% caracterizou como ruim ou péssimo esses aspectos da assistência (Tabela 2).

No modelo logístico multinominal ajustado, as variáveis independentes apoio empático e respeitosos dos profissionais de saúde, privacidade no local do parto, clareza de informações recebidas pela mulher, tempo disponível para fazer perguntas e receber explicações e presença de acompanhante durante o trabalho de parto e parto apresentaram associação positiva e significante com a avaliação realizada pelas mulheres do cuidado recebido durante o parto e nascimento (Tabela 3).

Além disso, quanto o melhor a opinião da mulher a respeito de cada uma dessas práticas, mais positiva foi sua avaliação do cuidado recebido. Este padrão de associação foi observado para todas as variáveis que caracterizavam o relacionamento da equipe de cuidadores com a parturiente. Como exemplo disso, em comparação com as mulheres que classificaram o apoio empático e respeitoso dos profissionais como excelente, aquelas que avaliaram como regular, ruim ou péssimo tiveram cerca de 47 vezes mais chances (OR = 46.81, IC = 20.65 - 106.12) de avaliar o cuidado recebido como regular e 257 vezes mais chances (OR = 257.14, CI = 66.22 - 998.46) de avaliar como ruim ou péssimo (Tabela 3).

Mulheres que não tiveram a presença de acompanhante durante o trabalho de parto e parto tiveram 3.51 (OR = 3.51, CI = 1:11–11:12) vezes a chance de avaliarem a assistência recebida como ruim ou péssima comparada com as que tiveram acompanhante em todos os momentos do parto (Tabela 5). As demais boas práticas da categoria A analisadas (livre dieta, liberdade de movimento, analgesia por métodos não farmacológicos, amamentação na sala de parto, contato pele-a-pele após o nascimento e acompanhante de livre escolha) não apresentaram associação estatística com o desfecho (Tabela 3).

Discussão:

Este estudo encontrou que uma boa relação estabelecida entre as mulheres e sua equipe de cuidados durante o trabalho de parto e nascimento é um fator decisivo para a avaliação positiva do atendimento recebido. A falta de associação com os aspectos mais objetivos do cuidado, tais como a realização de certos procedimentos, pode ser devido ao fato de estes terem menor relevância no processo de avaliação pelas mulheres. Corroborando com essa ideia, muitos estudos têm relatado que aspectos mais subjetivos do cuidado, geralmente relacionados à forma como a equipa médica interage com a mãe, influenciam mais na avaliação positiva do cuidado do que as práticas relacionadas com os mais objetivos da assistência [16,17].

Hodnett (2002), em sua revisão sistemática, encontrou quatro fatores associados à satisfação das mulheres com a assistência recebida no parto: as expectativas da gestante em relação ao parto, a quantidade e qualidade do suporte recebido pela parturiente da equipe médica, a qualidade da relação paciente e equipe médica e envolvimento nas decisões a respeito do parto. Segundo a autora, esses quatro fatores parecem sobrepor aos fatores idade, situação socioeconômico, cor de pele, preparação para o parto, o ambiente físico, a dor, imobilidade e intervenções médicas. Portanto, o comportamento e a relação médico e paciente parecem ter mais peso e estarem mais associados à satisfação do que as outras variáveis [17].

Somado a isso, as informações e explicações recebidas durante o trabalho de parto e parto são importantes na boa avaliação da assistência recebida, pois auxiliam na autonomia e no protagonismo dessa mulher em relação ao parto. Se ela não é informada de como está o progresso do trabalho de parto e de quais procedimentos serão realizados, não tem como participar das escolhas, ficando, consequentemente, passiva frente à assistência. Estudos demonstram que a mulher se sentir protagonista de seu parto, no controle do que é feito, está associado a altos graus de satisfação com cuidado recebido no trabalho de parto e parto [18,19].

Outro aspecto relevante que esse estudo traz é a importância da presença do acompanhante durante todo o trabalho de parto e parto para a avaliação da qualidade do cuidado realizada pela mulher. Esse fato é corroborado por outros estudos que também relatam a importância, para as mulheres, da presença do acompanhante [20-22].

Outra questão importante para debate é a discrepância encontrada entre a baixa prevalência das boas práticas na assistência ao trabalho de parto e parto e a alta proporção de avaliação positiva pelas puérperas desse cuidado recebido. Esse resultado é discrepante, pois, por um lado temos uma assistência ao parto normal de baixa qualidade e, por outro, temos a grande maioria das puérperas avaliando positivamente esse cuidado. Cabe aqui, refletir sobre quais motivos que podem estar levando as mulheres a avaliarem positivamente uma assistência que está distante de ser aquela recomendada pelo MS e OMS.

Uma das possíveis causas para esse *gap* seria a falta de informação que as gestantes têm a respeito do que seria um parto de qualidade. Estudos apontam que o pré-natal na região sudeste não fornece informações sobre o direito ao acesso às boas práticas na assistência ao trabalho de parto e parto normais [23,24]. Por causa disso, grande parte das mulheres não conhecem seus direitos e os procedimentos que compõem o cuidado ideal. Sendo assim, como poderiam avaliar, ter senso crítico frente a algo que desconhecem? Esse fato é apontado como um dos limites desse tipo de estudo, pedir para pacientes avaliarem a assistência quando eles, em geral, desconhecem o padrão ideal [6].

Somado a essa questão da pouca informação, as expectativas que as mulheres têm em relação ao parto também influenciam a forma como ela irá avaliar essa assistência [25,26]. Em seu estudo sobre as expectativas e experiências das mulheres em relação ao parto, Dias e Deslandes (2006) encontraram relatos de violência verbal, abandono e demora do atendimento, alegando que muitas puérperas classificaram o atendimento recebido como bom apenas por não terem sofrido nenhum tipo de violência [27]. Portanto, pode ser que as expectativas de grande parte das mulheres sejam tão baixas que, um pouco que recebam, as fazem avaliar positivamente essa assistência.

Outro fator que pode estar associado a essa discrepância entre a qualidade da assistência e a avaliação feita pelas puérperas é a tendência, relatada na literatura, de as mulheres avaliarem mais positivamente a assistência recebida ao trabalho de parto e parto do que realmente foi [3]. Essa tendência é denominada por Teijlingen, et al (2003) como viés de gratidão (*Gratitude Bias*). Segundo esses autores, esse seria um viés que permearia e dificultaria todos os estudos sobre a avaliação e satisfação das puérperas com a assistência recebida nas maternidades. É como se as mulheres não pudessem avaliar negativamente a assistência recebida, pois consideram um ato de ingratidão frente ao desfecho positivo do parto [6].

Além disso, ao se pensar nas implicações dos resultados deste estudo, algumas outras questões metodológicas precisam ser levadas em consideração. Em primeiro lugar, devido à baixa prevalência de mulheres em algumas categorias de avaliação do atendimento recebido, algumas OR’s obtiveram um grande intervalo de confiança (IC), fato que pode comprometer a precisão dessas medidas de associação. Em segundo lugar, a ausência de qualquer variável de controle relacionada às expectativas das mulheres em relação ao atendimento recebido durante o trabalho de parto e parto influencia na análise mais profunda das associações.

Em contrapartida, o fato de a avaliação realizada pelas mulheres do cuidado recebido durante o trabalho de parto e parto ter sido mensurada fora do hospital e, em média, um ano após o parto, é um ponto forte desse estudo. Isso porque, este fato provavelmente resultou na diminuição do viés de gratidão. Estudos nessa temática recomendam que esse tipo de pergunta deve ser feito fora do ambiente hospitalar, pois as mulheres podem se sentir envergonhadas e com medo de represálias por parte da equipe de saúde se essa pergunta for feita ainda no hospital. Somado a isso, o senso crítico das mulheres em relação à assistência recebida tende a aumentar com o tempo [3,6]. Somado a isso, até onde sabemos, este é o primeiro estudo desse tipo no Brasil.

Conclusão:

Esta pesquisa encontrou que as boas práticas de característica mais subjetiva: privacidade no local do parto, tempo disponível para fazer perguntas e obter explicações, clareza das informações recebidas, apoio empático e respeitoso dos profissionais, contato pele a pele e presença do acompanhante durante o trabalho de parto parecem ter maior peso para uma avaliação positiva da qualidade da assistência ao parto para as mulheres. Esse resultado mostra a importância da relação entre a equipe de cuidadores e parturiente para uma experiência positiva do parto.

Nosso estudo não encontrou associação entre a avaliação realizada pelas puérperas e as boas práticas relacionadas com os aspectos objetivos do cuidado: analgesia por métodos não farmacológicos, dieta livre, livre movimentação, início da amamentação na sala de parto e contato pele a pele imediatamente após o nascimento. Este fato pode ser explicado pelo viés de gratidão, falta de informação das mulheres e baixa expectativa em relação a assistência ao trabalho de parto e parto. No entanto, não podemos ignorar a possibilidade de que, talvez, os aspectos subjetivos da assistência ao parto tenham mais relevância para as mulheres. Fato esse encontrado não apenas em nosso estudo, mas também em outras pesquisas sobre a temática.

A relação entre a equipe de cuidadores e a parturiente se mostra, portanto, de grande impacto para a forma como está ela irá vivenciar o processo do trabalho de parto e parto. Uma equipe atenciosa, acolhedora, com uma escuta qualificada e atenciosa pode auxiliar na melhoria da qualidade da assistência, proporcionando uma experiência mais positiva da parturição. Portanto, este estudo aponta para a necessidade de se investir na formação de profissionais de saúde, com o objetivo de desenvolver e aprimorar essas qualidades e habilidades nas equipes de cuidadores.

**Lista de abreviações:**

MS – Ministério da Saúde

OMS- Organização Mundial de Saúde

HIV- Human Immunodeficiency Virus

ABEP - Associação Brasileira de

OR - Odds Ratio

IC - Intervalo Confiança

**Conflito de interesse:**

Os autores declaram que não terem conflito de interesse.

**Contribuição dos autores:**

MLB concebeu a hipótese e o desenho do estudo, realizou a análise e discussão dos dados e elaborou o manuscrito. MMTF e SGNG trabalharam na discussão dos resultados e contribuíram para escrita do manuscrito. Todos os autores revisaram e aprovaram a versão final.

**Agradecimentos:**

Este trabalho foi financiado pelo Conselho Nacional de Desenvolvimento Científico e Tecnológico, pelo Departamento de Ciência e Tecnologia, Secretaria de Ciência, Tecnologia e do Ministério da Saúde, pela Escola Nacional de Saúde Pública da Fundação Oswaldo Cruz (INOVA Projeto) e pela Fundação de Amparo à Pesquisa do Estado do Rio de Janeiro. Gostaríamos de agradecer aos pesquisadores Edwin van Teijlingen e Ann-Kristin Sandin-Bojö pelas excelentes revisões do manuscrito.

**Referência:**

1. Donabedian A. Explorations in quality assessment and monitoring, Vol. 1. Ann Arbor: Health Administration Press, 1980.

2. Pittrof R, Campbell OMR, Filippi VGA. What is quality in maternity care? An international perspective. Acta Obstet Gynecol Scand. 2002; 81:277–283.

3. Waldenström U, Hildingsson I, Rubertsson C, Rådestad I. A negative birth experience: prevalence and risk factors in a national sample. Birth Berkeley Calif. 2004; 31:17–27.

4. Solomon RC, Stone LD. On ‘positive’ and ‘negative’ emotions. Journal for the Theory of Social Behaviour. 2002; 32: 417–435.

5. Farquhar M, Camilleri-Ferrante C, Todd C. Continuity of care in maternity services: women’s views of one team midwifery scheme. Midwifery. 2000; 16:35–47.

6. Van Teijlingen ER, Hundley V, Rennie A-M, Graham W, Fitzmaurice A. Maternity satisfaction studies and their limitations: “What is, must still be best.” Birth Berkeley Calif. 2003; 30:75–82.

7. Wilde- Larsson, B., Larsson, G., Kvist., L, Sandin- Bojö, AK. Women’s opinions on intrapartal care: development of a theory-based questionnaire *Journal of Clinical Nursing.* 2010; 19: 1748-1760.

8. Hotimsky SN, Rattner D, Venancio SI, Bógus CM, Miranda MM. Childbirth as I see it.... or the way I wish it was? Expectations of pregnant women towards. Cad Saúde Pública. 2002; 18:1303–1311.

9. Wilde-Larsson, B., Sandin-Bojö, AK., Starrin, B, Larsson, G. Birth giving women’s feelings and perceptions of quality of intrapartal care: a nationwide Swedish cross-sectional study. *Journal of Clinical Nursing.* 2011; 20: 1168-1177.

10. World Health Organization (WHO). Care in the normal birth: a pratical guide. 1996. <http://www.who.int/maternal_child_adolescent/documents/who_frh_msm_9624/en/> accessed 15^th^ Jun, 2014.

11. BRASIL, Ministério da Saúde, Secretaria de Políticas de Saúde. Manual Prático para implementação da rede cegonha. Brasília, DF: O Ministério, 2011. <http://webcache.googleusercontent.com/search?q=cache:dckFM7kGqhYJ:www.saude.mt.gov.br/arquivo/3062+&cd=1&hl=pt-BR&ct=clnk&gl=br> accessed 10th Jun, 2014.

12. De Vasconcellos MTL, Silva PLN, Pereira APE, Schi-lithz AOC, Souza Junior PRB, Szwarcwald CL. Sampling design for the Birth in Brazil: National Survey into Labor and Birth. Cad Saúde Pública. 2014; 30 Suppl:49-58.

13. Leal MC, Pereira APE, Domingues, RMS, Filha, MMT, Dias MAG, Nakamura-Pereira M, Bastos, MH, Gama SGN. Obstetric interventions during labor and childbirth in Brazilian low-risk women. Cad. Saúde Pública. 2014: 30 Sup l.1: S17-S32.

14. Dahlen HG, Tracy S, Tracy M, Bisits A, Brown C, Thornton C. Rates of obstetric intervention among low-risk women giving birth in private and public hospitals in NSW: a population-based descriptive study. BMJ Open. 2012; 2(5): 1-8.

15. ABEP: Associação Brasileira de Empresas de Pesquisa (ABEP). 2010. <http://www.abep.org/> accessed 15^th^ Jun, 2014.

16. Sawyer A, Ayers S, Abbott J, Gyte G, Rabe H, Duley L. Measures of satisfaction with care during labour and birth: a comparative review. BMC Pregnancy Childbirth. 2013; 13:108.

17. Hodnett ED. Pain and women’s satisfaction with the experience of childbirth: a systematic review. Am J Obstet Gynecol. 2002; 186(5 Suppl Nature):S160–172.

18. Tingstig C, Gottvall K, Grunewald C, Waldenström U. Satisfaction with a modified form of in-hospital birth center care compared with standard maternity care. Birth Berkeley Calif. 2012; 39:106–114.

19. Fair CD, Morrison TE. The relationship between prenatal control, expectations, experienced control and birth satisfaction among primiparous women. Midwifery. 2012; 28:39–44.

20. Carraro TE, Knobel R, Radünz V, Meincke SMK, Fiewski MFC, Frello AT, da Silva Martins M, Lopes CV, Berton, A. Cuidado e conforto durante o trabalho de parto e parto: na busca pela opinião das mulheres. Texto Contexto Enferm. 2006; 15:97–104.

21. Séguin L, Therrien R, Champagne F, Larouche D. The components of women’s satisfaction with maternity care. Birth Berkeley Calif. 1989; 16:109–113.

22. Hodnett ED, Gates S, Hofmeyr GJ, Sakala C. Continuous support for women during childbirth. Cochrane Database Syst Rev. 2012; 1: 2-17.

23. Carvalho DS de, Novaes HMD. Avaliação da implantação de programa de atenção pré-natal no Município de Curitiba, Paraná, Brasil: estudo em coorte de primigestas. Cad Saúde Pública. 2004; 20 Sup 2: S220-S230.

24. Puccini RF, Pedroso GC, Silva EMK, Araújo NS, Silva NN. Prenatal and childbirth care in an area in Greater Metropolitan São Paulo. Cad Saúde Pública. 2003; 19:35–45.

25. Christiaens W, Bracke P. Assessment of social psychological determinants of satisfaction with childbirth in a cross-national perspective. BMC Pregnancy Childbirth. 2007; 7: 26-35.

26. Green JM, Renfrew MJ, Curtis PA. Continuity of care: what matters to women? A review of the evidence. Midwifery. 2000; 16:186–196.

27. Dias MAB, Deslandes SF. Expectativas sobre a assistência ao parto de mulheres usuárias de uma maternidade pública do Rio de Janeiro. Cad Saúde Pública. 2006; 22:2647–55.

| Table 1 |  |  |
| --- | --- | --- |
| Características sociodemográficas e obstétrica das puérperas e avaliação realizada | | |
| pelas mulheres do cuidado recebido. Pesquisa "Nascer no Brasil", sudeste, 2011-2012. |  |  |
|  | n | % |
| **Avaliação do cuidado** |  |  |
| Excelente | 1529 | 37.3 |
| Bom | 2137 | 52.1 |
| Regular | 310 | 7.2 |
| Ruim/Péssimo | 126 | 3.4 |
| **Paridade** |  |  |
| Multípara | 2114 | 50.9 |
| Primípara | 2041 | 49.1 |
| **Tipo de parto** |  |  |
| Vaginal | 3055 | 73.5 |
| Forceps / Vácuo Extrator | 173 | 4.2 |
| Cesariana | 928 | 22.3 |
| **Idade (anos)** |  |  |
| 12 - 19 | 952 | 22.9 |
| 20 - 34 | 2947 | 71.0 |
| 35 or more | 255 | 6.1 |
| **Cor de pele** |  |  |
| Branca | 1416 | 34.1 |
| Preta | 397 | 9.6 |
| Parda | 2275 | 54.8 |
| Amarela | 66 | 1.1 |
| **Escolaridade** |  |  |
| ES comp/mais | 180 | 4.4 |
| EM comp | 1809 | 43.7 |
| EF comp | 1259 | 30.4 |
| EF incomp | 891 | 21.5 |
| **Condição econômica** |  |  |
| A + B | 886 | 21.5 |
| C | 2501 | 60.5 |
| D + E | 741 | 18.0 |
| **Tipo de pagamento** |  |  |
| Privado | 466 | 11.2 |
| Público | 3689 | 88.8 |

|  |  |  |  |  |  |  |  |  |  |  |
| --- | --- | --- | --- | --- | --- | --- | --- | --- | --- | --- |
| Tabela 2 |  |  |  |  |  |  |  |  |  |  |
| Prevalência das boas práticas de acordo com OMS. Pesquisa "Nascer no Brasil", sudeste, 2011-2012. | | | | | |  |  |  |  |  |
| **Boas práticas OMS** | **Excelente** | | **Bom** | | **Regular** | | **Ruim** | | **Péssimo** | |
|  | n | % | n | % | n | % | n | % | n | % |
| Apoio empático/respeitoso | 1751 | 42.1 | 1868 | 44.9 | 352 | 8.6 | 68 | 1.6 | 116 | 2.8 |
| Privacidade no local do parto | 1717 | 41.3 | 1955 | 47.1 | 323 | 7.8 | 75 | 1.7 | 85 | 2.1 |
| Clareza das informações recebidas | 1589 | 38.2 | 1880 | 45.2 | 448 | 10.8 | 124 | 3.1 | 114 | 2.7 |
| Tempo disponível para perguntas/esclarecimentos | 1139 | 27.4 | 2230 | 53.7 | 546 | 13.1 | 140 | 3.4 | 101 | 2.4 |
|  | **Sim** | | **Não** | |  |  |  |  |  |  |
| Acompanhante de livre escolha | 2907 | 90.7 | 298 | 9.3 |  |  |  |  |  |  |
| Dieta livre | 1412 | 34.5 | 2744 | 65.5 |  |  |  |  |  |  |
| Livre movimentação | 1871 | 45.0 | 2285 | 55.0 |  |  |  |  |  |  |
| Uso de método não farmacológico | 2618 | 37.5 | 1538 | 62.5 |  |  |  |  |  |  |
| Contato pele a pele | 1413 | 34.1 | 2732 | 65.9 |  |  |  |  |  |  |
| Aleitamento na sala de parto | 2010 | 48.6 | 2139 | 51.6 |  |  |  |  |  |  |
|  | **No** | | **Partial** | | **Yes** | |  |  |  |  |
| Presença do acompanhante | 962 | 23.1 | 2192 | 52.8 | 1008 | 24.1 |  |  |  |  |
|  |  |  |  |  |  |  |  |  |  |  |

| **Tabela 3** |  |  |  |  |  |  |
| --- | --- | --- | --- | --- | --- | --- |
| *Odds ratio* (OR) brutos e ajustados* para as boas práticas na assistência ao trabalho de parto e parto da avaliação do cuidado recebido pelas puérperas. Pesquisa "Nascer no Brasil", região sudeste, 2011-2012. | | | | | | |
| **Boas práticas OMS** | Avaliação do cuidado recebido pelas puérperas** | | | | | |
|  | Bom | | Regular | | Ruim/Péssimo | |
|  | OR bruto (IC) | OR ajustado (IC) | OR bruto (IC) | OR ajustado (IC) | OR bruto (IC) | OR ajustado (IC) |
| **Apoio empático/respeitoso** |  |  |  |  |  |  |
| Excelente | 1.00 | 1.00 | 1.00 | 1.00 | 1.00 | 1.00 |
| Bom | 4.13 (2.91- 5.87) | 4.03 (2.90-5.59) | 3.15 (1.43 6.90) | 3.31 (1.48-7.39) | 11.17 (3.06 40.82) | 10.77 (2.65-43.75) |
| Regular/Ruim/Péssimo | 6.43 (3.72-11.13) | 6.53 (3.73-11.42) | 40.63 (19.38-85.18) | 46.81 (20.65-106.12) | 217.00 (59.71 788.65) | 257.14 (66.22-998.46) |
| **Privacidade no local do parto** |  |  |  |  |  |  |
| Excelente | 1.00 | 1.00 | 1.00 | 1.00 | 1.00 | 1.00 |
| Bom | 4.03 (2.95-5.50) | 3.87 (2.85-5.24) | 7.59 (3.69-15.63) | 8.56 (3.88-18.91) | 16.38 (3.41-78.73) | 16.97 (3.54-81.29) |
| Regular/Ruim/Péssimo | 5.21 (2.78-9.76) | 5.16 (2.73-9.74) | 44.56 (19.57-101.48) | 55.12 (21.88-138.83) | 182.96 (30.95-1081.73) | 217.18 (33.73-1398.43) |
| **Clareza das informações recebidas** |  |  |  |  |  |  |
| Excelente | 1.00 | 1.00 | 1.00 | 1.00 | 1.00 | 1.00 |
| Bom | 3.64 (2.86-4.65) | 3.44 (2.69-4.39) | 3.39 (1.88-6.10) | 3.38 (1.82-6.30) | 12.31 (3.34-45.36) | 10.96 (2.94-40.88) |
| Regular/Ruim/Péssimo | 6.46 (3.42-12.21) | 6.02 (3.29-10.99) | 26.25 (14.40-47.86) | 58.98 (15.10-55.66) | 88.26 (21.47-362.83) | 88.88 (21.45-368.24) |
| **Tempo disponível para perguntas/esclarecimentos** |  |  |  |  |  |  |
| Excelente | 1.00 | 1.00 | 1.00 | 1.00 | 1.00 | 1.00 |
| Bom | 3.04 (2.18-4.25) | 2.93 (2.08-4.12) | 5.14 (1.97-13.44) | 5.03 (1.90-13.32) | 3.69 (1.48-9.21) | 3.71 (1.49-9.28) |
| Regular/Ruim/Péssimo | 6.17 (4.19-9.07) | 5.78 (3.95-8.46) | 33.63 (13.71-82.47) | 33.77 (13.08-87.19) | 23.27 (8.27-65.42) | 25.74 (8.79-75.32) |
| **Dieta livre** |  |  |  |  |  |  |
| Sim | 1.00 | 1.00 | 1.00 | 1.00 | 1.00 | 1.00 |
| Não | 0.96 (0.62-1.42) | 1.00 (0.66-1.53) | 0.88 (0.57-1.35) | 0.89 (0.54-1.45) | 0.81 (0.39-1.68) | 0.76 (0.35-1.64) |
| **Livre movimentação** |  |  |  |  |  |  |
| Sim | 1.00 | 1.00 | 1.00 | 1.00 | 1.00 | 1.00 |
| Não | 0.87 (0.60-1.25) | 0.95 (0.65-1.38) | 0.92 (0.59-1.43) | 0.98 (0.62-1.55) | 0.59 (0.32-1.11) | 0.61 (0.32-1.16) |
| **Uso de métodos não farmacológicos** |  |  |  |  |  |  |
| Sim | 1.00 | 1.00 | 1.00 | 1.00 | 1.00 | 1.00 |
| Não | 0.83 (0.61-1.12) | 0.87 (0.63-1.20) | 1.18 (0.72-1.95) | 1.24 (0.74-2.07) | 0.97 (0.49-1.93) | 0.99 (0.51-1.91) |
| **Contato pele a pele** |  |  |  |  |  |  |
| Sim | 1.00 | 1.00 | 1.00 | 1.00 | 1.00 | 1.00 |
| Não | 1.28 (0.85-1.93) | 1.29 (0.84-1.99) | 1.25 (0.63-2.47) | 1.26 (0.61-2.60) | 2.28 (0.97-5.35) | 2.28 (0.93-5.56) |
| **Aleitamento na sala de parto** |  |  |  |  |  |  |
| Sim | 1.00 | 1.00 | 1.00 | 1.00 | 1.00 | 1.00 |
| Não | 0.93 (0.69-1.25) | 0.99 (0.72-1.35) | 1.08 (0.59-1.97) | 1.10 (0.59-2.06) | 0.81 (0.44-1.47) | 0.78 (0.43-1.42) |
| **Acompanhante de livre escolha** |  |  |  |  |  |  |
| Sim | 1.00 | 1.00 | 1.00 | 1.00 | 1.00 | 1.00 |
| Não | 1.36 (0.81-2.28) | 1.28 (0.76-2.15) | 0.98 (0.25-3.86) | 0.83 (0.22-3.08) | 2.52 (0.86-7.35) | 2.02 (0.64-6.41) |
| **Presença de acompanhante** |  |  |  |  |  |  |
| Sim | 1.00 | 1.00 | 1.00 | 1.00 | 1.00 | 1.00 |
| Às vezes | 0.96 (0.61-1.51) | 0.98 (0.61-1.57) | 0.85 (0.37-1.92) | 0.89 (0.41-1.92) | 1.27 (0.47-3.41) | 1.12 (0.40- 3.14) |
| Não | 1.32 (0.77-2.26) | 1.28 (0.72- 2.30) | 2.38 (0.88-6.43) | 2.44 (0.99-6.03) | 3.51 (1.07-11.50) | 3.51 (1.11-11.12) |
| IC: intervalo de 95% de confiança. |  |  |  |  |  |  |
| * Modelos ajustados pelas variáveis sociodemográficas (condição econômica, escolaridade, tipo de financiamento do parto), paridade e tipo de parto. | | | | |  |  |
| ** Categoria de referência: Excelente |  |  |  |  |  |  |
| Nota: Devido às baixas proporções de puérperas, as categorias Regular, Ruim e Péssimo, em todas as boas práticas, foram agrupadas. |  |  |  |  |  |  |
