# Supplementary material for: Good practices according to WHO’s recommendation for normal labor and birth and women’s assessment of the care received: the “birth in Brazil” national research study, 2011/2012
Source: Reprod Health. 2016 Oct 17;13(Suppl 3):124. doi: 10.1186/s12978-016-0233-x (PMC5073993; doi:10.1186/s12978-016-0233-x)
Supplement: Additional file 2: — Peer review. (PDF 407 kb) [file 12978_2016_233_MOESM2_ESM.pdf]

Referee's comments to the authors– this sheet WILL be seen by the author(s) and published with the article

Title	Association between good practices for normal labor and birth and women's assessment of the care received: a cohort study
Author(s)	Márcia Leonardi Baldisserotto, Mariza Miranda Theme Filha and Silvana Nogueira da Gama
Referee's name	Ann-Kristin Sandin Bojö

When assessing the work, please consider the following points, where applicable:

1. **Is the question posed by the authors new and well defined?**
The question is interesting and to my knowledge new.

2. Are the methods appropriate and well described, and are sufficient details provided to replicate the work?
The Method is not well described and at the moment not possible to follow and it is not possible to replicate the study.

3. Are the data sound and well controlled?
It is not possible to judge the data from what is described in the Method section.

4. Does the manuscript adhere to the relevant standards for reporting and data deposition?
The Tabels are easy to follow and gives a good description of the Results.

5. Are the discussion and conclusions well balanced and adequately supported by the data?
6. Do the title and abstract accurately convey what has been found?
7. Is the writing acceptable?
No. See coments in the manuscript

Please make your report as constructive and detailed as possible in your comments so that authors have the opportunity to overcome any serious deficiencies that you find and please also divide your comments into the following categories:

- Major Compulsory Revisions (which the author must respond to before a decision on publication can be reached)
- Minor Essential Revisions (such as missing labels on figures, or the wrong use of a term, which the author can be trusted to correct)
- Discretionary Revisions (which are recommendations for improvement but which the author can choose to ignore)

Where possible please supply references to substantiate your comments.

When referring to the manuscript please provide specific page and paragraph citations where appropriate.

Comment [U@K1]: Pages are missing in the manuscript

General comments: The subject is interesting – to study the association between good practices for normal birth and women's assessment of care. However , the ¨story line¨ in the Background and Method is unclear and the way references are used are difficult to follow. Please see comments in the manuscript.

Major compulsory revisions:

Please see comments in the manuscript.

Minor essential revisions:
Please see comments in the manuscript.

Referee's comments to the authors– this sheet WILL be seen by the author(s) and published with the article

Title	Association between good practices for normal labor and birth and women's assessment of the care received: a cohort study
Author(s)	Márcia Leonardi Baldisserotto, Mariza Miranda Theme Filha and Silvana Nogueira da Gama
Referee's name	Edwin van Teijlingen

When assessing the work, please consider the following points, where applicable:

1. Is the question posed by the authors new and well defined?
2. Are the methods appropriate and well described, and are sufficient details provided to replicate the work?
3. Are the data sound and well controlled?
4. Does the manuscript adhere to the relevant standards for reporting and data deposition?
5. Are the discussion and conclusions well balanced and adequately supported by the data?
6. Do the title and abstract accurately convey what has been found?
7. Is the writing acceptable?

Please make your report as constructive and detailed as possible in your comments so that authors have the opportunity to overcome any serious deficiencies that you find and please also divide your comments into the following categories:

- Major Compulsory Revisions (which the author must respond to before a decision on publication can be reached)
- Minor Essential Revisions (such as missing labels on figures, or the wrong use of a term, which the author can be trusted to correct)
- Discretionary Revisions (which are recommendations for improvement but which the author can choose to ignore)

Where possible please supply references to substantiate your comments.

When referring to the manuscript please provide specific page and paragraph citations where appropriate.

General comments:

Interesting study with clear research question and relatively straightforward outcomes. Methods could be a little clearer.

Major compulsory revisions:
It is not clear of the authors collected the original data or whether they were independent of the ".. research data collected in the *Nascer no Brasil* survey, a national hospital-based study consisting of mothers and their newborns, conducted between February 2011 and October 2012 in Brazil." If the authors got the data set to do the analysis, this study is really secondary analysis. Which is fine, but as a method it has strengths and weaknesses which the authors need to acknowledge properly in the Methods. I think this was the case as reference 17 highlighting the survey methods has different authors
If the authors did all the data collection themselves then rewrite the first sentence: "We used research data collected in the *Nascer no Brasil* survey, a national hospital-based study consisting of mothers and their newborns, conducted between February 2011 and October 2012 in Brazil." To something like: "We were part of the team conducting the *Nascer no Brasil* survey, a national hospital-based study in Brazil consisting of mothers and their newborns, between February 2011 and October 2012.

(continue on the next sheet)

Continued:
Minor essential revisions:

The sentence "**Background:** The World Health Organization recommends good practices for the conduct of uncomplicated labor and birth, with the aim of improving the quality and assessment by women of maternity care" in the Abstract needs and extra 'of' : "**Background:** The World Health Organization recommends good practices for the conduct of uncomplicated labor and birth, with the aim of improving the quality **of** and assessment by women of maternity care."

Background typo in "offered [1,2] he opinion.." should this be: offered [1,2] the opinion …"
Throughout the text 'et al.' should be with a full stop after 'al.' as it is an abbreviation. The authors do this inconsistently at the moment
The sentence "..Teijlingen et al. (2003) called this trend "gratitude bias"." Should read: "van Teijlingen et al. (2003) called this trend "gratitude bias".

The authors have a slightly confusing way of referring to their quantitative questionnaire survey. "Interviews of postpartum …" interviews are qualitative research tools, here you have survey participants and questionnaire studies that were conducted….

The section on ethics suggest all women could read and write: "all participating women signed an informed consent form." Is this correct? Is there a possible bias here re. illiterate women?

As part of the Discussion, just above the Conclusions I expected to find a paragraph with the strengths and weaknesses of this study. These may include as positive being first study of its kind in Brazil and as weakness to use of secondary data (i.e. once you have no control over as they have already been collected).

There is no reference number in the sentence "In one systematic review, Hodnett (2002) found four factors.."

Table 2 has 'Excelente' instead of English 'excellent'

Discretionary revisions:

Be consistent in the use of numbers in the text, e.g. 1 year and two telephone interviews: "about 1 year after birth, in which women were asked to give a second assessment of the care received. Because of losses of sample subjects during follow-up via the two telephone ..." I would suggest one to ten in words and 11 upwards in numbers

Table headings are a little bit long

Supplement Editor comments:

- An English edition of the paper is needed. We advise you to use the service of Edanz http://www.edanzediting.com/ . We are having a good experience about the quality of the English edition of this initiative. Another initiative that you can use is AuthorAid : http://www.authoraid.info/en/

- In references not coming from journals please provide the link and the date that you accessed such link.
- Discussion section is too long. Please try to condense such section. A section about strengths and weakness of your study is needed.
